# Programming of Multicellular Patterning with Mechano‐Chemically Microstructured Cell Niches

**DOI:** 10.1002/advs.202204741

**Published:** 2023-03-30

**Authors:** Peter L. H. Newman, Queenie Yip, Pierre Osteil, Tim A. Anderson, Jane Q. J. Sun, Daryan Kempe, Maté Biro, Jae‐Won Shin, Patrick P. L. Tam, Hala Zreiqat

**Affiliations:** ^1^ ARC Training Centre for Innovative Bioengineering The University of Sydney Sydney 2006 Australia; ^2^ Embryology Research Unit Children's Medical Research Institute Sydney 2145 Australia; ^3^ School of Medical Science Faculty of Medicine and Health The University of Sydney Sydney 2006 Australia; ^4^ Swiss Cancer Research Institute (ISREC) School of Life Sciences Ecole Polytechnique Fédérale de Lausanne Lausanne 1005 Switzerland; ^5^ EMBL Australia Single Molecule Science Node School of Medical Sciences UNSW Sydney 2052 Australia; ^6^ Department of Pharmacology and Regenerative Medicine University of Illinois at Chicago Chicago IL 60607 USA

**Keywords:** micropatterning, multicellularity, pluripotent stem cells, tissue models, tissue patterning

## Abstract

Multicellular patterning of stem‐cell‐derived tissue models is commonly achieved via self‐organizing activities triggered by exogenous morphogenetic stimuli. However, such tissue models are prone to stochastic behavior, limiting the reproducibility of cellular composition and forming non‐physiological architectures. To enhance multicellular patterning in stem cell‐derived tissues, a method for creating complex tissue microenvironments endowed with programmable multimodal mechano‐chemical cues, including conjugated peptides, proteins, morphogens, and Young's moduli defined over a range of stiffnesses is developed. The ability of these cues to spatially guide tissue patterning processes, including mechanosensing and the biochemically driven differentiation of selected cell types, is demonstrated. By rationally designing niches, the authors engineered a bone‐fat assembly from stromal mesenchyme cells and regionalized germ layer tissues from pluripotent stem cells. Through defined niche‐material interactions, mechano‐chemically microstructured niches enable the spatial programming of tissue patterning processes. Mechano‐chemically microstructured cell niches thereby offer an entry point for enhancing the organization and composition of engineered tissues, potentiating structures that better recapitulate their native counterparts.

## Graphical abstract

Stem‐cell‐derived tissue models exhibit stochastic cellular compositions with malformed architectures. To enhance such tissues, a method for orthogonal programming of material microstructure with biochemicals across a range of Young's moduli is developed. Through designed niche‐material interactions the authors can spatially program tissue patterning processes, enabling the enhancement of tissue organization and composition, potentiating structures that better recapitulate their native counterparts.

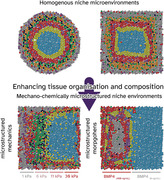

## 1 Introduction

Tissue histogenesis proceeds by integrating mechanical, chemical, and topological information. Such “mechano‐chemical” information directs the signals through which diverse cell types, tissues, and organs emerge.^[^ ^1^, ^2^ ^]^ While leveraging developmental processes can yield tissues and organ models with complex structures,^[^ ^3^, ^4^, ^5^, ^6^ ^]^ current approaches rely on exogenously administered signaling factors and biomaterials with bulk properties. Such stimuli may preclude the generation of tissues with proper architecture and physiological functions. Further, “self‐organizing” mechanisms elicited by globally administered exogenous stimuli are prone to stochastic behaviors, generating tissue and organ models that do not reproducibly capture the structure‐function relationships in native tissues and organs.

These limitations can be partially overcome by confining cells to micropatterns that topologically constrain cell signaling for tissue patterning.^[^ ^3^, ^7^, ^8^ ^]^ Accordingly, approaches that integrate micropatterning with the delivery of local biochemical cues have been developed.^[^ ^4^, ^9^ ^]^ However, the use of this approach alone lacks the capacity for simultaneous control over the complex multimodal cues, including topological, mechanical,^[^ ^10^, ^11^, ^12^ ^]^ and biochemical^[^ ^13^, ^14^, ^15^, ^16^, ^17^ ^]^ stimuli present during tissue histogenesis.

Towards better control over histogenic processes, printing approaches have been developed to control cell functions, including microstructured growth factors,^[^ ^18^ ^]^ 3D‐patterning of nerve growth factors in hydrogels,^[^ ^19^ ^]^ chemically microstructured materials,^[^ ^20^ ^]^ mechanically microstructured materials,^[^ ^21^ ^]^ and mechano‐chemically microstructured materials.^[^ ^22^ ^]^ Such printing methods traditionally involve the extrusion of a biopolymer ink or the direct extrusion of cells. Extrusion printers can produce materials with discrete properties through the sequential extrusion of different materials, either from separate print cartridges, each loaded with different bioinks,^[^ ^23^, ^24^, ^25^, ^26^, ^27^, ^28^ ^]^ or through mixing solutions before their extrusion through a single nozzle.^[^ ^22^, ^29^ ^]^ Alternatively, printed tissues can be obtained indirectly by extruding and differentiating stem cells such as hiPSCs.^[^ ^30^, ^31^, ^32^ ^]^ While this approach permits generating of macroscale organoids with improved reproducibility, these models still rely on self‐organizing processes, limiting their size and tissue complexity and, in turn, constraining their acquisition of higher‐level cellular function

Photolithography is an alternative 3D printing method that uses light to selectively polymerize a material from a photoresist. This method offers technical solutions to some obstacles faced when extrusion‐printing complex materials. For example, photolithographic printing methods have generated materials with nanoscale features,^[^ ^33^ ^]^ a feat yet to be achieved using extrusion printing. One recent advanced demonstration of a photolithographic method for fabricating complex chemically microstructured materials describes a novel enzymatic chemistry for the spatiotemporal photopatterning and release of epidermal growth factor ,^[^ ^34^ ^]^ and subsequent increases in cell proliferation as a result.

Critically, while bioengineering methods such as 3D printing improve control over multicellular systems, these works have not demonstrated spatially reproducible control over local signaling, including cell attachment, mechanosensing, local interactions with transcription factors, and multicellular tissue patterning (see Supporting Information). Literature generating complex multicellular tissue systems is limited to controlling the size and shape of tissue progenitors,^[^ ^8^, ^30^, ^32^, ^35^ ^]^ with the delivery of any signaling factors administered globally or with bespoke microfluidic devices.^[^ ^4^, ^9^ ^]^

In the present work, we address these limitations using a printing method. We show multimodal material cues can define the shape and size of tissue progenitor colonies while additionally providing defined local mechanical and chemical cues to guide the structure (shape and multicellularity) of the cells they support. Our printing method can precisely control a material's local mechanical and chemical microproperties over the physiological range, including Young's Modulus and concentrations of small and large biomacromolecules. We demonstrate control over cellular‐scale mechanosensing and the regionalized differentiation of selective cell types. Printed cell niche materials with specific microstructured properties can support the generation of stem‐cell‐derived tissue constructs, including a bone‐fat‐assembly from stromal progenitors. Our studies revealed a novel role for niche mechanics in directing germ layer tissue patterning where mechanics direct tissue organization reminiscent of germ layer differentiation and a material‐mediated morphogen signaling that recapitulates localized signaling of mesendodermal differentiation. The systematic nature of the method and high level of control to arbitrarily define complex mechanochemically structured cellular environments enables the bottom‐up study of the minimal cues required to support tissues, adding the capacity to explore the roles of mechano‐chemical signaling and advance our understanding of how complex multimodal signals are integrated to direct tissue histogenesis.

## 2 Results

### 2.1 Printing Microstructured Niches with Mechano‐Chemical Flow Lithography

Photolithographic printing methods can fabricate materials with complex properties by changing the composition of a photoresist during printing. Here, photoresists of different compositions are serially injected or flowed through a polymerization volume,^[^ ^20^ ^]^ giving the name of this method: flow lithography (FL).^[^ ^36^ ^]^ This approach overcomes the technical hurdles of extrusion printing methods by using separate subsystems for solution injection, mixing, and placement/polymerization, whereby materials with structured properties can be fabricated with a resolution primarily limited by a polymerization volume of focused light, the so‐called “spot‐size” – see Supporting Information for the complete methods and characterization of the printing technologies.

**Figure 1:**
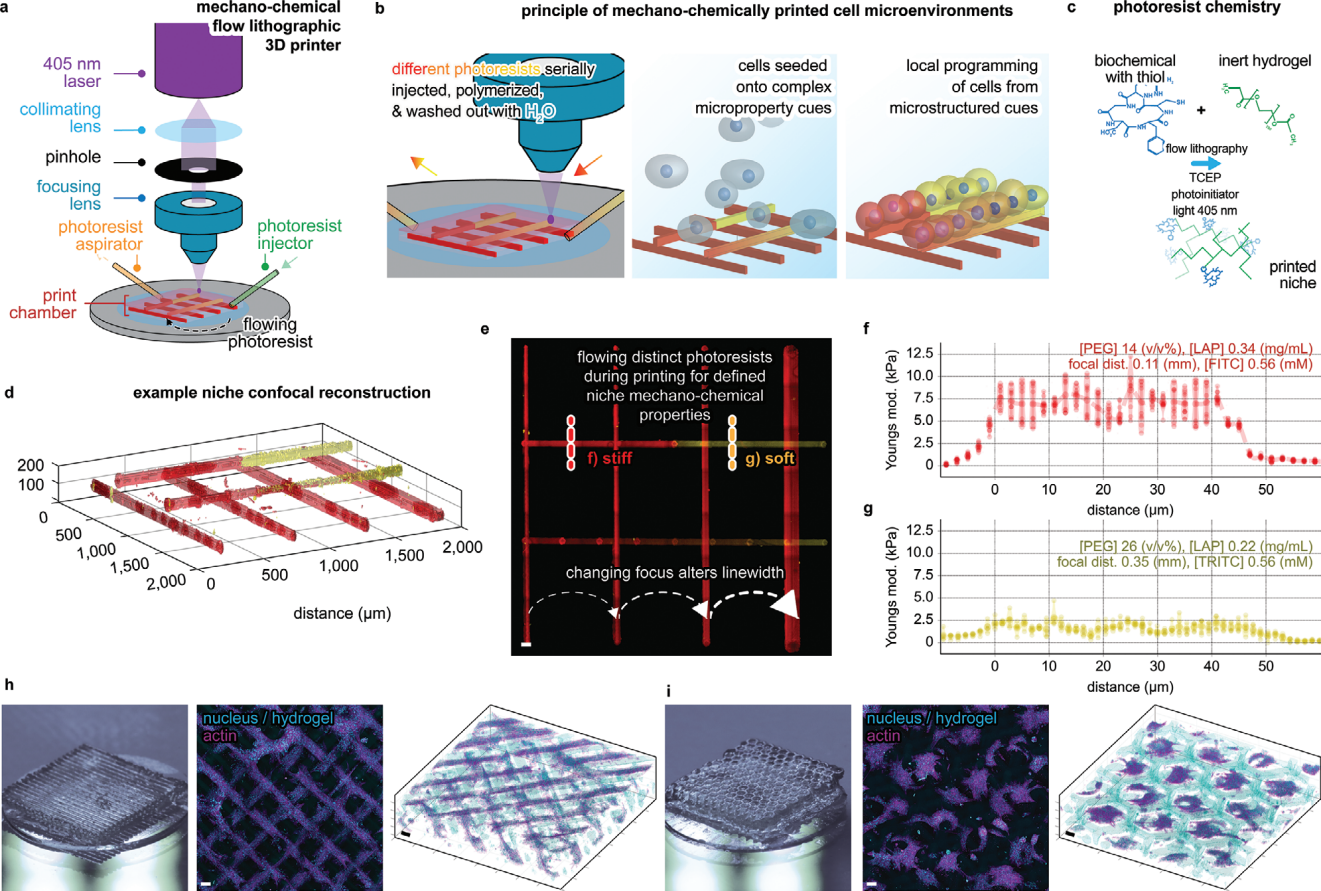
Mechano‐chemical flow lithographic (MCFL) printing of microstructured niches. a) Components of the MCFL 3D printer. b) Stepwise fabrication processes to print synthetic cell niche environments. c) Photoresist chemistry used. d) Confocal image of an example niche with microstructured properties, including changes to linewidth, mechanics (Young's modulus), and chemical microproperties (concentration of the fluorophores FITC and TRITC). e) Maximum intensity projection of the 3D confocal data, scale bar 50 µm. Dotted red and orange lines annotate the profile of force spectroscopy in (f,g). Young's modulus across filaments. Fabrication variables are shown at the top of the graphs for physiologically f) stiff (7.5 kPa, red, TRITC) and g) soft (2.5 kPa, orange, FITC) segments. h,i) Actin (magenta) and hydrogel (cyan) stained ADSCs (primary human adipose‐derived stromal cells) cultured over 3D niche with (h), “stacked‐logs” or i) “offset‐honeycomb‐layers” architecture. Macro lens photography (left) is shown together with MIP (middle) and 3D confocal renders (right), scale bars 200 µm.

**Figure 2:**
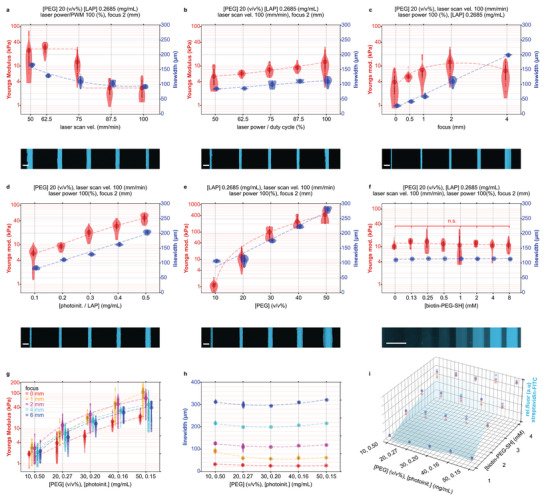
Model for printing mechano‐chemically microstructured properties. a–f) Microstructured niche properties were printed using a model that relates printer and photoresist variables to Young's modulus (left axis, red) and linewidth (right axis, blue). Independent variables are indicated in the subfigure base above microscopy of printed filaments with streptavidin‐FITC conjugate. Gamma correction is applied to subset (f) for improved visibility. Fabrication variables that remain constant are enumerated at the top of each subset. Scale bars, 200 µm. g–i) The optimized variable state‐space. Discrete material properties can be interpolated from the optimized state space to generate niches with microstructured properties. g) The relationship between the unified photoinitiator‐monomer concentration (horizontal axis) and focus (shown in different colors) on Young's modulus and h) linewidth. i) The effect of photoinitiator‐monomer (horizontal axis), Biotin‐PEG‐SH concentration (into‐page), and focus (shown in different colors) on the bound thiol‐ene conjugate. n.s. – *p* > 0.05 by one‐way ANOVA with Tukey post‐hoc tests. Violin plots show mean with 1^st^/3^rd^ quartile lines. Splines‐of‐best‐fit is plotted to highlight trends. Throughout (g), data points are offset on the horizontal axis to minimize overlap of the concurrently shown dependent variable Young's modulus.

To explore the potential of FL printing, we custom‐built a printer allowing the fabrication of complex cell niche environments by flowing photoresists of variable biochemical and polymeric composition through a chamber during printing (**Figure** **1** and Figures S1—S3, Supporting Information). Our photoresist was selectively polymerized using a 405 nm laser, with changes to the laser's focus controlling the resolution and size of printed structures (Figure 1b). The minimal linewidth printable with this technique was shown to be 7 µm (Figure S4, Supporting Information). The photoresist is composed of a bioinert hydrogel monomer (polyethylene glycol diacrylate), a photoinitiator (lithium phenyl(2,4,6‐trimethylbenzoyl)phosphinate [LAP]^[^ ^37^ ^]^), and biochemicals, which can include any peptide, protein, or morphogen with a thiol‐functional‐group available from cysteine peptide moieties (Figure 1c–e and Figure S5, Supporting Information). Following laser‐induced photocleavage of the photoinitiator, the hydrogel monomer polymerizes alongside a thiol‐ene bioconjugation reaction that can covalently crosslink biochemicals to the otherwise bioinert material, specifying bioactivity. Relatively stiff (Figure 1b,f) or soft (Figure 1b,g) Young's moduli are achieved by respectively increasing or decreasing the monomer and photoinitiator concentration (Figures 1f,g and 2d,e). Coordinating the above, mechano‐chemical flow lithography (MCFL) can print 3D architectures that support cell attachment and growth (Figure 1h,i).

To print materials with independently tunable mechano‐chemical properties, we developed a model that relates the MCFL fabrication variables to the printed linewidth, Young's modulus, and the concentration of conjugated thiol‐ene biochemical of the printed hydrogels. We characterized six variables affecting material properties, including three variables controlled by the printer (laser scan velocity, focus, and laser power) and three photoresist variables (the concentration of photoinitiator, monomer (polyethylene glycol ‐ PEG), and bioconjugate Biotin‐PEG‐SH, a model biochemical with free thiol group ‐ SH) (**Figure** **2**a–f). Decreased light exposure (Figure 2a,b), photoinitiator (Figure 2d), and monomer concentrations (Figure 2e) decreased both Young's modulus and the linewidth of prints, consistent with a lowered rate of polymerization due to the reduced light absorption and consequentially lower photoinitiator cleavage and monomer conversion (see notes on photopolymerization in Supporting Information, Figures S6 and S7, Supporting Information). The diameter of the conic angle of laser transmittance linearly correlated to changes in linewidth (Figure 2c). We further characterized the effect of changing Biotin‐PEG‐SH concentration on material Young's modulus and linewidth. We found that Biotin‐PEG‐SH inclusions up to 8 mM did not significantly alter Young's modulus or linewidth (Figure 2f).

The relationship between independent variables that simplifies the number of independent printing variables while still exhibiting physiologically relevant material properties was characterized (Figure 2g–i). This simplification was achieved by fixing the laser scan velocity to 100 mm min^−1^ and the laser power to 100%, as well as by unifying the two variables of photoinitiator and monomer concentration to a single variable by changing the concentration of one as a function of the other (Experimental Section, Equation 2). These simplifications left three independent variables: focus, bioconjugate concentration, and the combined concentration of photoinitiator–monomer. Niches with microstructured properties could then be printed by interpolating the relevant fabrication variables from the simplified state‐space and discretizing the printing processes (Figure 2g–i, see Experimental Section for additional details). We demonstrated this approach by printing filaments with all permutations of either increasing or decreasing Young's moduli, linewidth, and relative bioconjugate fluorescence (Figure S8, Supporting Information). This method allowed the characterization of a state‐space and subsequent interpolation of materials properties for Young's moduli between 2–20 kPa, biochemical additives to 4 mM, and linewidths from 40 to 300 µm (Figure 2g–i).

### 2.2 Niche‐Programmed Cell Attachment, Spreading, and Mechanosensing

**Figure 3:**
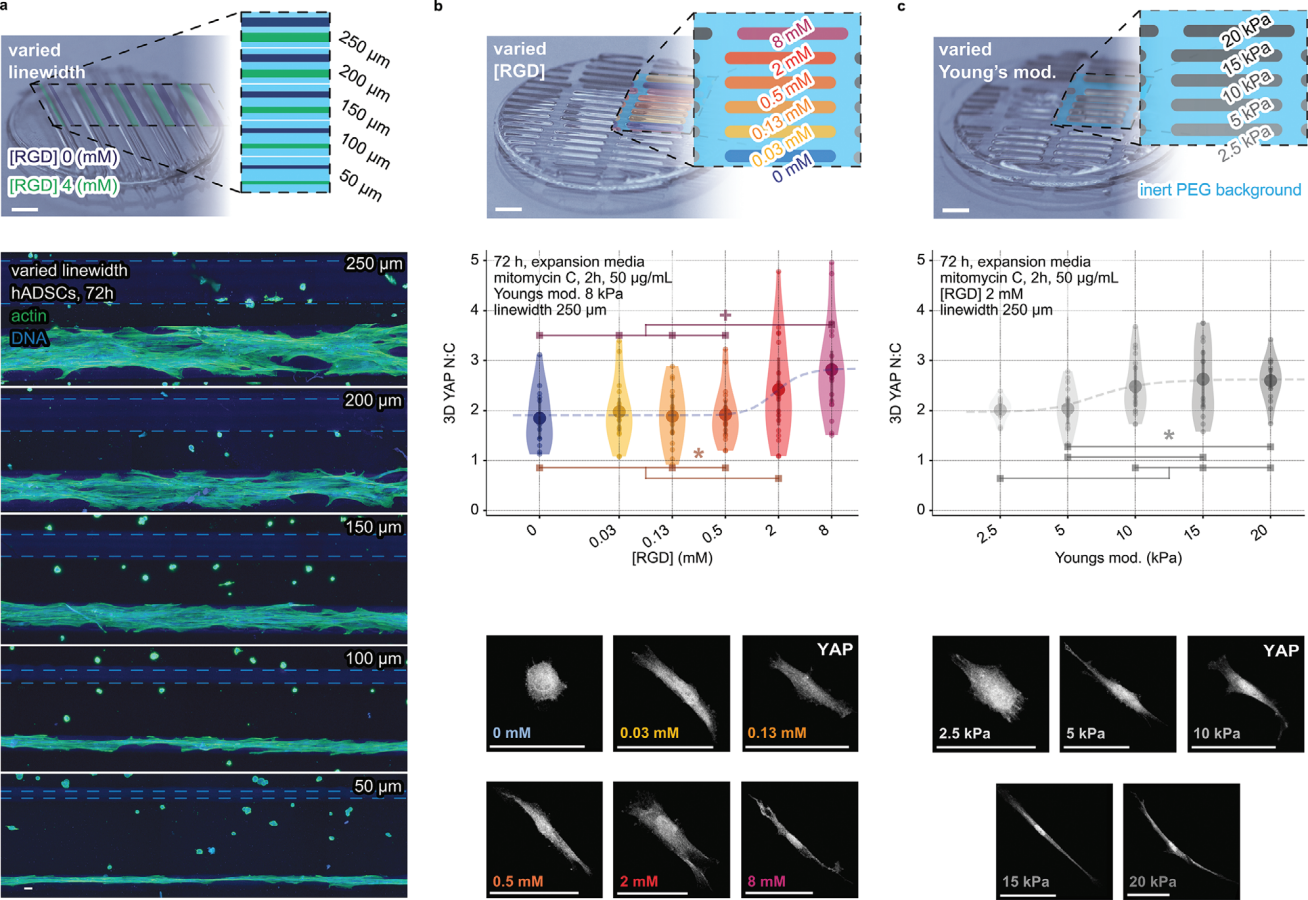
Niche‐programmed cell attachment, spreading, and mechanosensing. The effect of microstructured niche properties, including the concentration of the peptide cell‐attachment ligand RGD and Young's modulus, is shown to regulate cell attachment, spreading, and mechanosensing. a) Printed filament pairs with 4 or 0 mM RGD, from 50–250 µm, with a fixed Young's modulus of 8 kPa. Representative confocal MIPs show actin (phalloidin, green) and nucleus (DAPI, blue). Gamma correction and dotted lines help improve the visibility of the zero RGD concentration dark blue filaments where no cells attach. Niche arrays with microstructured b) chemical (RGD), and c) mechanical (Young's modulus) properties are shown alongside the quantification and representative confocal MIPs of the nuclear to cytoplasmic ratio (N:C) of the biomolecular mechanostat YAP in hADSCs at 72 h as imaged on their respective microproperty regions. Violin plots show mean with 1^st^/3^rd^ quartile lines. Sigmoidal best‐fit lines are plotted alongside results to highlight trends. Scale bars, 50 µm, except macro lens images of MCFL arrays at figure top = 1 mm. Schematic overlays of material properties introduce color coding for ease of reading. *, + denotes *p* < 0.05, 0.10 by one‐way ANOVA with Tukey post‐hoc tests.

We explored if microstructured niche properties could program cellular‐scale attachment and mechanosensing changes. To test this, we printed niches with a physiologically moderate Young's modulus of 8 kPa^[^ ^38^ ^]^ in parallel filaments between 50–250 µm wide (**Figure** **3**a and Table S2, Supporting Information). On culturing multipotent human adipose‐derived stromal cells (hADSCs) for 72 h, cells selectively attached to regions printed with the cell attachment peptide RGD (cyclo(Arg‐Gly‐Asp‐d‐Phe‐Cys)), displaying increasing spreading over filaments of larger linewidth. We next explored if microstructured RGD concentrations could regulate cell attachment and spreading in a dose‐dependent fashion. Niches structured with six regions of RGD concentrations between 0–8 mM were printed at a moderate Young's modulus (8 kPa) and a fixed linewidth (250 µm) (Table S2, Supporting Information). We showed that the extent of cell attachment and spreading correlated with the RGD concentration (Figure 3b), demonstrating that microstructured niche chemistry can define local changes to cell attachment and spreading.

We postulated that microstructured niches could spatially program complex cell functions, such as cellular‐scale changes to mechanosensing. We replicated works exploring mechanosensing,^[^ ^39^ ^]^ using our method of microstructured mechanical cues, by measuring the localization of YAP/TAZ, a protein that acts as a mechanostat when comparing the relative distribution of YAP/TAZ in the nucleus (N) and the cytoplasm (C), or the YAP N:C ratio, wherein a high N:C ratio indicates a mechanically active cell with relatively high intracellular force.^[^ ^39^ ^]^ As previously used, we printed niches with six chemically microstructured RGD concentrations, as well as niches with mechanical microstructure, with five regions of differing Young's moduli between 2.5–20 kPa, corresponding to a range from physiologically soft‐to‐stiff (Figure 3c and Table S2, Supporting Information). hADSCs cultured on these niches displayed changes to YAP N:C ratio in a sigmoidal response to changes in the microstructured concentration, revealing that local cell mechanosensing could be regulated through the microstructure of the underlying chemical (RGD, Figure 3b and Figure S9, Supporting Information) and mechanical (Young's moduli, Figure 3c) properties.^[^ ^39^, ^40^ ^]^ The lower threshold of YAP N:C correlated to a low concentration of RGD and soft Young's modulus, while for high concentrations of RGD and stiff Young's modulus, an upper threshold of YAP N:C was observed. This is the first demonstration of a method to define niches with complex mechano‐chemical microproperties that elicit a local mechanosensing response via HIPPO signaling,^[^ ^41^ ^]^ a critical regulator of tissue patterning, morphogenesis, and organ growth.

### 2.3 Niche‐Programming of a Bone‐Fat Construct

**Figure 4:**
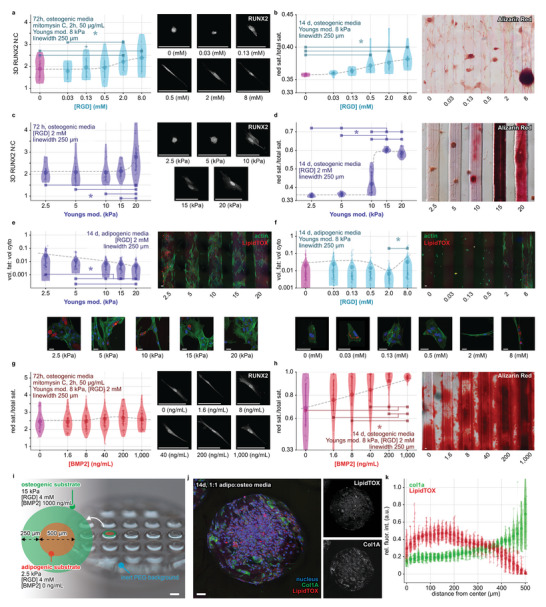
Niche‐programmed patterning of a bone‐fat‐assembly. Niche properties are systematically studied to engineer a bone‐fat construct assembled from local material‐mediated interactions. a,b) Osteogenic differentiation of hADSCs on chemically (RGD) microstructured niches. a) Immunofluorescent staining and quantification of RUNX2 and b) quantification and representative widefield images of Alizarin Red stained bone mineralization. c,d) Osteogenic differentiation over mechanically (Young's modulus) microstructured properties, revealed by RUNX2 and Alizarin Red staining. e,f) Adipogenic differentiation over mechanically (Young's modulus) and chemically (RGD) microstructured properties, with representative MIPs and quantification of LipidTOX stained fat volume per cytoplasmic volume (vol. fat:cyto). g,h) Osteogenic differentiation on microstructured BMP2, revealed by RUNX2 and Alizarin Red stains. i–k) Bone‐fat‐assemblies that mimic the osteon with a central adipogenic region and a peripheral osteogenic region. i) Printed MCFL bone‐fat niche array with overlaid text detailing material properties j) Confocal MIP image shows nuclear (blue), brightfield (grey), Col1A (green), and LipidTOX (red) channels, with grayscale insets showing Col1A and LipidTOX channels, gamma‐corrected for ease of visibility. k) Quantification of the normalized pixel intensity for Col1A and LipidTOX data across replicates. Scale bars 100 µm, except *d* = 1 mm. Violin plots show mean with 1^st^/3^rd^ quartile lines. Splines‐of‐best‐fit‐lines are plotted alongside results to highlight trends. Scale bars 100 µm, except for *i* = 1 mm. *, + denotes *p* < 0.05, 0.10 by one‐way ANOVA with Tukey post‐hoc tests.

We explored the application of mechano‐chemically structured cell niche environments for spatially programming tissue patterning in multilineage structures. Given the upstream role of cellular mechanosensing in cell fate decisions,^[^ ^10^, ^39^, ^42^ ^]^ we sought to deliver local microstructured mechano‐chemical cues to drive osteogenic and adipogenic differentiation of multipotent stromal cells,^[^ ^10^, ^39^ ^]^ in a structure resembling an osteon. Osteogenesis was assessed by measuring the N:C ratio of RUNX2, an essential transcriptional regulator for the commitment of stromal cells to osteoblastic and early osteogenic lineages,^[^ ^43^ ^]^ and by visualizing mineralized bone deposition by Alizarin Red staining. Enhanced osteogenesis was revealed by elevated RUNX2 N:C ratio and enhanced mineralization, as correlated with increasing concentrations of RGD (**Figure** **4**a,b) and stiffer Young's moduli (Figure 4c,d). Adipogenesis, assessed as the volumetric ratio of fat‐to‐total‐cytoplasmic volume (fat:cyto) (Figure 4e,f), was correlated with mechanically soft (Young's modulus) or peptide‐enriched (high RGD) regions. We showed that the highest fat:cyto ratio was associated with mechanical properties with 8 mM RGD and a low Young's modulus (with ≈7‐fold increase at 2.5 kPa relative to 20 kPa). Further, we tested niches with varying concentrations of BMP2, which is known to influence osteogenesis. Cells cultured over regions microstructured with BMP2 exhibit scaling of RUNX2 N:C, with increasing RUNX2 expression for concentrations up to 200 ng mL^−1^, though decreasing at 1000 ng mL^−1^. Mineralization, assayed by Alizarin Red activity, was enhanced with increasing BMP2 concentrations, showing that high BMP2 concentrations accelerate osteogenesis (Figure 4g,h).

We next sought to program multilineage functions of hADSCs in a defined structure. To elicit concurrent osteo‐ and adipogenic signaling, we printed niches with separate mechano‐chemical microproperties for osteo‐ and adipogenic differentiation (Figure 4a–h), with a centralized soft adipogenic region of a high concentration of RGD surrounded by a stiffer osteogenic region with a high concentration of BMP2 and RGD (Figure 4i). hADSCs seeded on the bone‐fat microstructured niche and cultured in a 1:1 adipogenic:osteogenic media for 14 days displayed concurrent osteogenesis (COL 1A‐expressing region, Figure 4j) and adipogenesis (high lipid content region, Figure 4j) over their respective microstructured domains (Figure 4k and Figure S10, Supporting Information).

Cells cultured over a local niche exhibit varied responses when juxtaposed with another niche with different properties. For example, in the BMP2 and RGD‐contained regions (Figure 4b,g,h, linewidth 250 µm, Young's modulus 8 kPa, RGD 2 mM, and BMP2 0 ng mL^−1^), mineralization varied over these regions, and adipogenesis on BMP‐free RGD and different Young's Modulus substrate (Figure 4e,f, linewidth 250 µm, Young's modulus 8 or 5/10 kPa, RGD 2 mM and BMP2 0 ng mL^−1^) also varied. This suggests that complex behaviors arise over niches, wherein local cell signaling is altered by cell‐cell feedback between cells on neighboring microstructures. A better understanding of complex signaling feedback is critical to recapitulating the complex interdependence between cell types. These findings underscore the importance of a system able to deconstruct localized controls and the need for a method to deconstruct these, such as in the present work.

While the multipotency of stromal‐derived cells such as hADSCs lacks rigorous evidence,^[^ ^44^, ^45^ ^]^ their use herein permits contextualization and comparison with seminal works that explore the relationship between material properties, mechanobiological, and multilineage functions.^[^ ^10^, ^39^, ^42^ ^]^ These results show local niches composed of defined mechano‐chemical cues can guide the adipogenic and osteogenic activity of hADSCs and coordinate the generation of a biologically relevant tissue model that exhibits structure‐function relationships.

### 2.4 Mechano‐Structured Niche Patterning of Germ Layer Tissues

**Figure 5:**
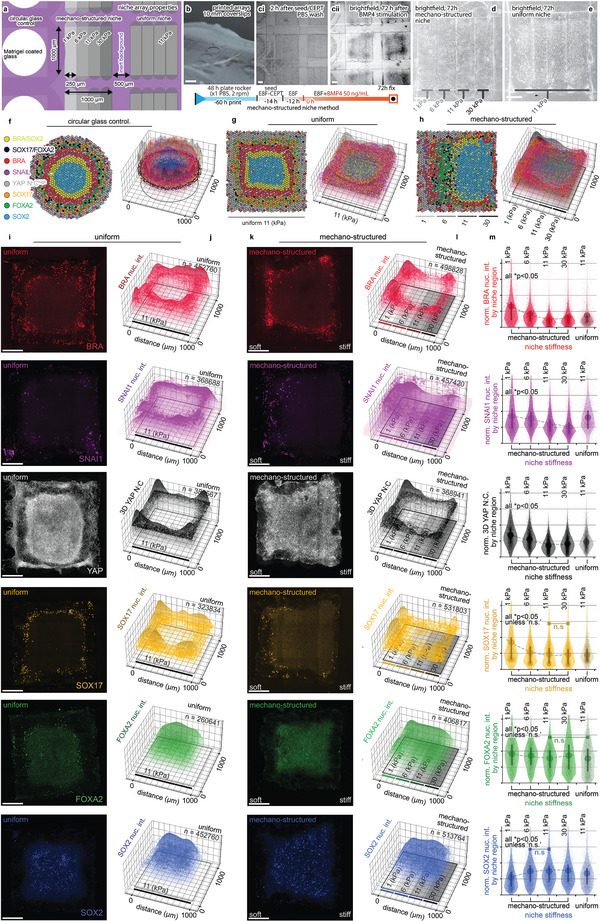
Microstructured niche mechanics recapitulate soft matrix and regionalizes tissue pattern. The patterning of hiPSCs cultured over circular control, uniform, and mechano‐structured niches. Radially symmetric marker expression is observed in uniform and circular control niches, compared with marker patterns over mechano‐structured niches, where mesoderm (BRA‐red, SNAI1‐purple) and endoderm (SOX17‐yellow, FOXA2‐green) are localized in niches of low Young's modulus. In contrast, ectoderm (SOX2+) cells are localized to niche regions of high Young's modulus. Data are collated at 72 h, except (c). Orientation across the figure is aligned (left‐to‐right) with soft‐to‐stiff mechano‐structures. a,b) Schematic and macro‐lens photography show coverslips with printed arrays of the three niche types with overlaid text indicating material properties. Scale bar, 1 mm. c) Brightfield image of arrays (see the overview of the experimental method and timeline in the top panel).  Brightfield image of d) mechano‐structured and e) uniform niches, with indicated mechanical properties. f–h) Schematic summary of marker expression alongside the pooled marker expression data shown as 3D scatter and surface plots overlaid on a representation of the corresponding niche. Each dot represents a single cell with size, height, and transparency scaled to the normalized fluorescent intensity. i,k) Representative confocal maximum intensity projections images for uniform and mechano‐structured niches, respectively. Scale bar, 200 µm. j,l) 3D scatter and surface plots for each marker overlaid on the corresponding niche. m) Violin plots compare the relative distribution and mean differences of uniform and mechano‐structured properties, including relative mean and 1^st^/3^rd^ quartile lines of replicate data mapped to respective material property regions. “n.s.” denotes *p* > 0.05 with all remaining group permutations *p* < 0.05 by one‐way ANOVA with Tukey post hoc tests.

During embryogenesis, changes in the mechanics of the extracellular matrix are critical for initiating mesodermal differentiation^[^ ^46^, ^47^, ^48^ ^]^ However, studies modulating mechanical heterogeneities in the embryonic niche remain unexplored in the context of human tissue patterning, a phenomenon that remains prohibitive to investigate. To explore the role of soft matrix mechanics in the differentiation of human germ layer tissues, we compare three niche environments; i) a 1000 µm diameter Matrigel‐coated‐glass control niche (circular control), ii) an MCFL printed square with uniform properties throughout (uniform niche), and iii) an MCFL square with graded mechano‐structure in Young's modulus (mechano‐structured niche) (**Figure** **5**a and Figure S11, Supporting Information). Cultures of hiPSCs were treated with BMP4 (50 ng mL^−1^ in Essential 8‐Flex, or E8F) for 72 h. Immunostaining of tissue markers for cell differentiation was then characterized using confocal microscopy and the StarDist segmentation tool^[^ ^49^, ^50^ ^]^ (Figure 5j,l and Figure S12, Supporting Information). Circular and square uniform niches replicated the center‐to‐periphery (radial) patterning of germ‐layer derivatives (Figure 5f–m, and Figures S13–S20, Supporting Information), consistent with graded signaling cascades in the radial dimension.^[^ ^3^, ^51^, ^52^ ^]^ Our findings on cells cultured on mechano‐structured niches exhibited variations in mesendoderm propensity along the graded mechano‐structure with cells populating low Young's modulus niches differentiated preferentially to BRA+, SNAI1+ mesoderm, and SOX17+/FOXA2+ double positive endoderm (Figure 5g–m and Figures S15, S16, S19, and S20, Supporting Information). In contrast, SOX2+ ectodermal cells populated regions of higher Young's modulus (Figure 5g–m and Figures S15, S16, and S19, Supporting Information). These findings are consistent with the association of mesodermal differentiation in the small‐molecule enhancement of cellular tension,^[^ ^7^, ^53^ ^]^ which is also reflected by high YAP N:C (Figure 5l,m, Figures S15 and S19, Supporting Information). Data presented in violin plots confirmed that mesodermal and endodermalcells populated niche regions with soft mechanics and ectodermal cells over stiffer mechanics (Figure 5m), with data at 48 h revealing a similar trend (Figure S15, Supporting Information). Mesoderm and endoderm cells were localized to regions with enhanced SNAI1 expression, indicative of active epithelial‐to‐mesenchymal‐transition (EMT)^[^ ^54^ ^]^ (Figure 5k–m and Figures S14, S15, and S17–S19). Differences in the marker regionalization between uniform and mechano‐structured niches highlight the impact of extracellular mechanics on germ layer differentiation, highlighting how mechano‐structured niche cues can be used to spatially program cell differentiation and thereby enhance the organization of engineered tissues (Figure S27, Supporting Information). Our findings are consistent with the notion that soft extracellular matrix may induce mesendodermal differentiation and EMT, reminiscent of this phenomenon in embryos,^[^ ^45^, ^46^, ^47^ ^]^ affirming that the mechanobiological attributes of the matrix play a role in guiding mesoderm differentiation.

### 2.5 Material‐Mediated Delivery of Localized Morphogens for Programming Tissue Patterning

**Figure 6:**
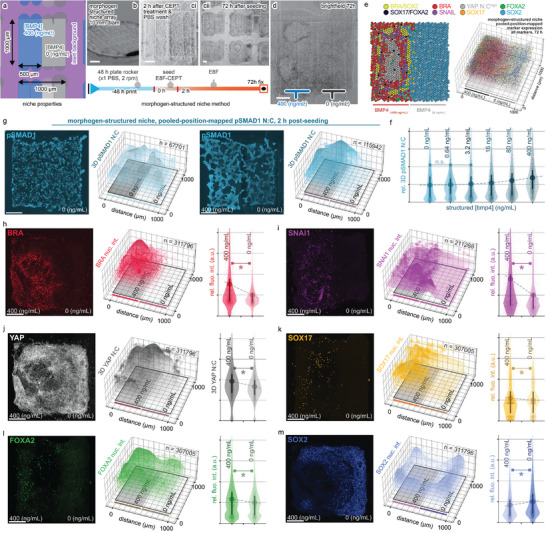
Material‐mediated BMP4 signaling programs tissue patterning, inducing foci of mesendoderm tissue. Patterning of hiPSCs cultured over morphogen‐structured niches. At 72 h, foci appear over domains with BMP4, shown as brightfield and confocal images. Densely packed mesoderm cells (BRA‐red, SNAI1‐purple, YAP ^high^‐gray) are surrounded by diffusely scattered endoderm cells (SOX17‐yellow, FOXA2‐green), whereas ectodermal (SOX2‐blue) cells are localized to domains without BMP4. Orientation: BMP4 containing domain (left), domain with no BMP (right). a) Schematic of niche array with indicated morphogen‐structured properties. b) Macro lens photography of array. Scale bar, 1 mm. c) Experimental timeline and brightfield imaging at c‐i) 2 h and c‐ii,d) 72 h. e) Schematic summary of marker expression alongside the pooled marker expression data shown as 3D scatter and surface plots overlaid on a representation of the corresponding niche, with each dot within the maps representing a single cell with size, height, and transparency scaled to the normalized fluorescent intensity. f) Violin plots of relative mean and distribution differences of the BMP4 signal transducer pSMAD1, in response to different concentrations of BMP4 in the morphogen‐structured niche at 2 h post‐seeding. 1^st^/3^rd^ quartile lines are shown. g) Distribution of pSMAD1 N:C at 2 h in morphogen‐structured domain with 400 ng mL^−1^ BMP4 (left) and domains without BMP4 (right). h–m) Representative confocal MIPs. Scale bar, 200 µm, 3D scatter/surface plots, and violin plots of marker expression on morphogen‐structured niches at 72 h. * denotes *p* < 0.05, “n.s.” denotes *p* > 0.05 by one‐way ANOVA with Tukey post‐hoc tests.

During development, histogenesis proceeds from locally secreted morphogens. We explored the functionality of MCFL niches for the local delivery of morphogens by printing morphogen‐structured square niches composed of two discrete domains, one with BMP4 and the other without (**Figure** **6**a and Table S2, Supporting Information). Unlike other micropatterns, morphogen‐structured niches do not require exogenous supplementation of factors to elicit cell differentiation (Figure 6b,c). Analysis of the distribution of pSMAD1 BMP4 signal transducer in the cells, measured as nuclear:cytoplasmic (N:C) ratio (Figure 6f,g, and Figures S22 and S23, Supporting Information), revealed that cells cultured over the BMP4‐structured regions exhibited an increased pSMAD1 N:C ratio indicating a positive response to the local BMP4 signal (Figure 6f,g). By 72 h, cells over the BMP4‐structured regions formed foci (Figure 6d,h–i and Figure S24, Supporting Information) of BRA+, SNAI1+ mesoderm cell scattered among SOX17+/FOXA2+ double positive endoderm cells (Figure 6f,h—m and Figures S25 and S26, Supporting Information). Mesoderm cells were densely packed in the high YAP N:C ratio core of the SNAI1+ foci (Figure 6h,l,m and Figure S24, Supporting Information).

This demonstration shows, for the first time, material‐mediated morphogen signaling centers and their application to spatially define cell differentiation and tissue patterning derived from locally divergent signaling. Differences in the pattern of marker expression between uniform and morphogen‐structured niches highlight how morphogen‐structured niche cues can be used to spatially program cell differentiation and thereby enhance the organization of engineered tissues (Figure 6e and Figure S27, Supporting Information).

## 3 Conclusion

Mechano‐chemically microstructured niches can program cell functions through defined niche‐material interactions. We demonstrate that mechanically and chemically microstructured niche properties can program multicellularity from localized cues, driving cell mechanosensing and biochemically mediated differentiation of stem/progenitor cells. This demonstration shows that material‐mediated morphogen signaling can spatially constrain multicellular tissue patterning, including germ layer differentiation of hiPSCs, and the formation of a bone‐fat assembly from stromal mesenchyme. This method overcomes a reliance on homogenous signaling as limited by bulk material properties or traditional globally administered media‐based exogenous morphogenetic cues,^[^ ^5^, ^6^ ^]^ and provides alternatives to non‐materials‐based strategies, including microfluidic systems,^[^ ^4^, ^9^ ^]^ microsurgical approaches,^[^ ^55^ ^]^ and optogenetics.^[^ ^56^ ^]^ Therefore, MCFL microstructured niches provide an entry point for better understanding the complex multimodal mechano‐chemical interactions that define cell and tissue behaviors, as well as for the engineering of multicellular tissue structures that better recapitulate the structure of their native counterparts.

Using mechano‐chemically microstructured niches complements current methods for generating complex stem‐cell‐derived tissue models. Expanding our approach in controlling localized signaling could enable the specification of tissues of divergent phenotypes and their later convergence and reintegration with complex multicellular interactions. The continued application of these niche systems has the potential to investigate unanswered questions in biology, such as how complex structure and function emerge; how the shape, size, and body coordinates of an organism are determined; and how mechanical and positional morphogenetic cues work in concert with morphogens and lineage determinants.

### 3.1 Limitation of this Study

One overlooked aspect of the present work is the impact of cell density on the derivation of complex multicellular tissues. Like topological constraints,^[^ ^3^ ^]^ cell density plays a key role in establishing the signaling gradients and feedbacks that determine cell behaviors and complex emergent tissue functions.^[^ ^57^ ^]^

Future work using such niches may extend to multi‐layer structures with increased complexity and topological constraints. Multi‐layer structures can be fabricated as per Figure 1h,i, using chemical photoabsorbers to simplify the printing of 3D structures^[^ ^58^ ^]^ or multiphoton lithography. As multiphoton photolithography can achieve sub‐diffraction‐limited resolutions,^[^ ^33^ ^]^ combining multiphoton methods with FL may enable printing materials with subcellular features and nanostructured properties.

The MCFL method could be advanced by adopting more specific conjugation chemistry. While our method demonstrates the function of complex biomolecules, next‐generation conjugation methods may improve biochemical specificity and function. Specifically, our method is limited when conjugating relatively complex macromolecules with numerous cysteine sites. Under these conditions, thiol‐ene conjugation occurs stochastically and can lead to the bioconjugate's loss of function. This limitation has been addressed with more reproducible chemistries, including enzymatic methods^[^ ^34^ ^]^ and specific high‐affinity non‐covalent binding chemistries.^[^ ^18^ ^]^ Further, such complex chemistries address the potential for the undesired cleavage and release of thiol‐ene‐bound growth factors that may otherwise occur. Additionally, during the generation of MCFL materials, the biological activity of a given biochemical must be considered. For example, the biological activity of some morphogens exhibits relatively short half‐lives as intrinsic to their resulting morphological function. In each case, the biological activity of a given biochemical factor must be determined in coordination with the MCFL protocol, which demands niche preparation, including printing, washing, and sterilization steps.

## 4 Experimental Section

### Custom‐Built MCFL 3D Printer

The MCFL printer was built from custom hardware and uses software developed by the authors. In‐depth details are available in the Supporting Information, listing directions for the up‐to‐date printer and a detailed printing method. Critical components included the high‐resolution *x*/*y* and *z* stages (V‐528.1AA /V‐528.1AB and M‐406 including corresponding controllers C‐413 and C863 from Physik Instrumente (PI) GmbH & Co. KG), as well as 405 nm diode laser (Cobolt 06‐01 Series 405 nm, fiber pigtailed, FC/APC). Various mechano‐optical components were used and purchased from Thor Labs (listed in Table S1, Supporting Information). A copy of the printer software is included in the Supporting Information, and up‐to‐date software can be obtained by contacting the lead authors. All niches were printed interpolating the state‐space shown in Figure 2g–I, with fixed laser power of 100% PWM of the 150 mW Cobolt 06‐01, and a scanning velocity of 100 µm min^−1^.

### Measurement of Structured Niche Microproperties

Niches were fabricated on acrylate coverslips using the geometries, photoresist, and printer variables as reported. The measurement of Young's modulus was completed via force spectroscopy using a JPK. NanoWizard Sense AFM mounted on Nikon Ti microscope. The device was fitted with the SuperCut quartz cantilever holder for liquid immersion and used with Bruker MLCT pyramidal cantilevers with stiffness calibrated using the thermal noise method. For force‐displacement curve generation, samples and AFM cantilever were submerged in ×1 PBS. The cantilever approach velocity was fixed to 0.5 µm sec^−1^ and terminated at a threshold force of 10 nN. Measurements were taken from three independent experimental replicates from at least four different printed‐niche replicates in each experiment. The Young's modulus was calculated from each force‐displacement approach curve using a custom fitting program written in MATLAB, with automated contact point determination and fitting for an 18° half‐angle conic section (Sneddon model, as per Bruker recommendation for MLCT pyramidal cantilevers), with sample‐thicknesses bottom‐effect cone‐correction as per Gavara et al.^[^ ^59^ ^]^ Data for force spectroscopic curves of AFM tip displacement against indentation force were rejected when discontinuities in the curves were present, corresponding to samples slipping and an inaccurate indentation. For the sample shown in Figure 1d–g, only two independent experimental replicates were fabricated, as this sample only served to illustrate how a mechano‐chemically microstructured niche material could be fabricated with the MCFL methodology. One replicate was mounted for confocal microscopy (Figure 1d,e), and the other was analyzed with force spectroscopy (Figure 1e,f). Linewidth and bioconjugation were measured using confocal microscopy. Linewidth was directly measured using Fiji‐ImageJ across three independent experimental replicates with quantification of the concentration of Biotin‐PEG‐SH measured indirectly by measuring the relative fluorescence of bound streptavidin‐FITC. Indirect measurement was used to prevent the photobleaching or free radical attack of fluorescent molecules during photopolymerization, which otherwise limited interpretation.

### Interpolation Method for Structured Niches Microproperties

Empirical data was tabulated pairing dependent and independent variables, including Young's modulus, linewidth, and the concentration of bioconjugate (Figure 2g–i), with the monomer–photoinitiator ($``\mathrm{PEG}/\mathrm{PI}{}^{\prime \prime }$ below), focus (shown as $``Z{}^{\prime \prime }$) and [Biotin‐PEG‐SH]. Using MATLAB (2020a), we then calculated the value of the independent fabrication variables of *PEG*/*PI* and *Z* after substitution of the desired Young's modulus (*E*_*des*._) and linewidth (*W*_*des*._) as per Equation (1) below.

(1) $$\begin{array}{ccc} 0 & = & {\left(\frac{E_{des.}-\frac{\sum \left(\tau *E_{em.}\right)}{\sum \tau }}{E_{des.}}\right)}^{2}+{\left(\frac{W_{des.}-\frac{\sum \left(\tau *W_{em.}\right)}{\sum \tau }}{W_{des.}}\right)}^{2},\;\;\mathrm{where}\\ \tau & = & \frac{1}{0.1\sqrt{2\pi }}e^{\frac{-1}{2}\left(\frac{\left|\frac{PEG/PI_{em.}-PEG/PI}{40}\right|+\left|\frac{Z_{em.}-Z}{6}\right|}{0.1}\right)} \end{array}$$

where, *PEG*/*PI*_*em*._ *Z*_*em*._ are vectors from the independent paired variables from the tabulated empirical dataset presented in Figure 2g,h, and are used to calculate the vector *τ* that is substituted into Equation (1), where a solution is obtained for the dependent paired variables *E*_*em*._,*W*_*em*._. The numbers 40 and 6 in Equation (1) represent the normalization range for *PEG*/*PI* and *Z* respectively, over which data is interpolated. Equation (1) is solved by finding the minimum of unconstrained multivariable functions. The relationship between *PEG*/*PI* is calculated as the positive real solution of Equation (2).

(2) $$0=\left|{\left(\frac{PEG/PI-50}{40}\right)}^{2}+{\left(\frac{PI-0.5}{0.35}\right)}^{2}\right|-1$$

### Cell Culture

hADSCs were cultured in expansion media: MesenPRO RS™ Basal Medium (Invitrogen) with the supplement of 2 mM l‐glutamine and MesenPRO RS Growth Supplement (Life Technology). hADSCs in passage 3 were used for all the studies. A 10 mm coverslip with printed niche arrays was placed in a 48‐well plate for culture on niches. Using a biosafety cabinet, niche arrays were washed twice with PBS before 12 min UV‐light sterilization. The medium was changed every 3 days except for studies examining YAP and RUNX2 nuclear translocation, where hADSCs were treated with mitomycin (10 µg mL^−1^) (Cayman Chemical, 11435) for 2 h, to inhibit proliferation, 24 h after seeding, after which media was replaced with expansion media. For YAP and RUNX2 translocation studies, cells were seeded at 3000 cells cm^−2^. For differentiation studies, cells were seeded at 6000 cells cm^−2^, osteogenic (Gibco, A1007001) and adipogenic media (Gibco, A1007201) were used as noted. Experiments using iPSCs were performed with the HPSI0314i‐hoik_1 hiPSC line, obtained from the Wellcome Sanger Institute with the help of Cell Bank Australia. For routine passaging, cells were passaged with ReLeSR™ and grown on hESC‐qualified Geltrex‐coated 6‐well plates. All experiments used Essential 8 Flex media (E8F), with supplements as listed, Human BMP‐4 Recombinant Protein (Gibco, Catalog # PHC9534). For cell culture of iPSCs on niches, a 10 mm coverslip with printed niche arrays was placed in a 48‐well plate. The niche arrays were washed for 48 h on a plate rocker in PBS before 12 min UV sterilization using a biosafety cabinet. hiPSC cells were dissociated with ReLeSR and pipetted into a single cell suspension with CEPT cocktail^59^ before seeding at 1 million cells per 300 µL per well, or 500k cells per 300 µL per well for morphogen‐structured niches. After 2 h, media was replaced with E8F, before 48 h differentiation in 500 µL of E8F supplemented with 50 ng mL^−1^ BMP4 as reported (PHC9534 Gibco). Media was then replaced daily. All cells tested negative for mycoplasma contamination. For hADSCs, routine PCR assay checks (LookOut® Mycoplasma qPCR Detection Kit, MP0040A‐1KT) were completed for mycoplasma contamination, for hiPSC mycoplasma was tested using the fluorescent kit, MycoAlertTM Mycoplasma Detection Kit, Catalog #: LT07‐318.

### Immunofluorescence Imaging and Image Quantification

For immunostaining, all solutions, except for those with dilute antibodies and fluorophores, were syringe filtered through 0.22 µm membrane filters (Merck Millipore SLGP033RS). Cells were fixed at room temperature with 4% PFA in ×1 PBS buffer for 10 min and then washed three times with PBS, followed by 12 min permeabilization at room temperature with 0.1 w v^–1^% Triton X‐100 in PBS. Samples were then incubated in a blocking buffer of 3% BSA, 3.75 mg mL^−1^ glycine, and 0.05% w v^−1^ Tween 20 in PBS for 1 h at room temperature. Primary antibodies were diluted in 1% BSA and 0.05% w v^−1^ Tween 20 in PBS and added for 2 h (SOX2, 1:400, #3579, CST; SOX17, 1:400, Af1924, R&D; BRA, 1:400, AF2085, R&D; SOX17, 1:300, OTI3B10, Thermo; pSMAD1/5, 1:800, 41D10, CST; SNAIL, 1:500, AF‐3639, R&D; YAP, 1:400, sc‐101199, SCBT; RUNX2 AF488, 1:200, sc‐390351; NANOG, 1:500, ab109250, abcam; TRA‐1‐60, 1:500, ab109884, abcam; OCT4, 1:500, ab109884, abcam; SOX2, 1:500, ab109884, abcam; SSEA4, 1:500, ab109884, abcam; FOXA2, 1:500, ab10822, abcam). For indirect immunostaining, samples were washed ×3 times with PBS and incubated for 2 h at room temperature with corresponding secondary antibody (*α*‐mouse. 488, 1:500, ab150077, abcam; *α*‐rabbit. 488,1:500, ab150077, abcam; *α*‐goat. 594, 1:500, ab150077, abcam; *α*‐rabbit. 647, 1:500, ab150075, abcam). Nuclear and actin counterstains were performed using Abcam iFluor conjugated phalloidin (ab176753, ab176759), and Hoechst 33342 at 0.1 µg mL^−1^ (Sigma, 14533) dilute in ×1 PBS and incubated for 30 min at room temperature. Following counterstain incubation, samples were washed an additional ×3 with PBS containing 0.05% w/v % sodium azide. For YAP and RUNX2 translocation studies, microscopy was completed on a Zeiss LSM 800 Confocal microscope using 63× Objective Plan‐Apochromat 63×/1.40NA Oil objective (with an in‐plane lateral resolution of 0.413–0.124 µm per pixel) and pinhole diameter of 1.0 AU and azimuthal resolution of 0.4 µm. Nuclear images from Hoechst staining were used to create masks that define a nuclear volume. hADSC cytoplasmic masks were defined from flood‐filled phalloidin stains, with the average fluorescent intensity of each volume calculated in MATLAB. Therein, the YAP/RUNX2 nuclear to cytoplasmic translocation ratio was determined as the ratio of the mean YAP/RUNX2 fluorescent saturation intensity of the nuclear volume divided by the fluorescent saturation intensity in the non‐nuclear cell cytoplasmic volume. In Figures 3b,c, and 4a,c, YAP/RUNX2 measurements of a total of at least 12 single cells per condition were pooled across three independent experimental replicates from at least four printed‐niche replicates. Representative images were selected according to their proximity to the mean data as calculated across all replicates. For imaging hiPSCs, we used a Zeiss LSM 800 Confocal microscope using a Plan‐Apo 20x/0.8NA. Using Hoechst nuclear marker, individual cell nuclei were segmented using the StarDist algorithm,^[^ ^49^, ^50^ ^]^ defining nuclear masks. Using the nuclei masks, the fluorescent intensity of each stain channel was calculated. Then the coordinates of each nucleus within the niches were calculated, allowing replicate data to be remapped to a single plot that showed the average position mapped immunostained expression of the markers with marker size, height, and opacity scaled proportionally to the fluorescent intensity of the nuclei (see Supporting Information for additional details). All experiments containing expression‐mapped markers were completed from at least three independent experimental replicates, except for experiments analyzing the effects of different concentrations of pSMAD1 (Figure 6e–g, Figures S20 and S21, Supporting Information) completed in two independent replicates.

### LipidTOX, CNA35, and Alizarin Red Staining of hADSC Differentiation

The differentiation of hADSCs was assayed toward adipogenic and osteogenic lineage in response to niche microproperties. All solutions listed below were syringe filtered through 0.22 µm membrane filters (Merck Millipore SLGP033RS). Alizarin Red (Sigma, A5533) staining was performed to examine the presence of mineralized deposits under osteogenic differentiation conditions. Samples were washed ×2 with PBS before fixation at room temperature in 4% PFA dilute in x1 PBS buffer for 10 min and then washed three times with PBS. Samples were then washed ×3 in Milli‐Q H2O before incubation with Alizarin Red stain for 5 min (9.6 mg mL^−1^ Alizarin Red at a pH of 4.2, adjusted with acetic acid). Following incubation, samples were washed ×5 with Milli‐Q H2O, followed by a further ×3 washes with ×1 PBS containing 0.05 w/v % sodium azide. The cell mineralization was examined with Alizarin Red staining and widefield color microscopy of materials following 14 days of culture. Cells were imaged using a Nikon Ni‐E microscope with a color DS‐Fi2 camera and Plan Apo Lambda 10×/0.45NA dry objective. The localization of osteogenesis over specified RGD and Young's modulus regions was quantified for each niche‐replicate sample. The mean red saturation was divided by the mean total saturation that combines red, green, and blue color components for the corresponding region of interest. For Alizarin Red Stains in Figure 4b,d,h,k, measurements were pooled across three independent experimental replicates from at least four printed‐niche replicates in each experiment for at least 12 total measurements per niche condition. Staining with LipidTOX Red Neutral Lipid Stain (Thermo H34476) was completed to quantify cell fat volume under adipogenic differentiation conditions. Samples were fixed at room temperature with 4% PFA in ×1 PBS buffer at pH 7.4 for 10 min and then washed three times with PBS, followed by 12 min permeabilization at room temperature with 0.1 w/v % Triton X‐100 in PBS. Samples were then incubated with LipidTOX Red Neutral Lipid Stain (diluted 1:800), Abcam iFluor conjugated phalloidin (1:200), and Hoechst 33342 at 0.1 µg mL^−1^ (Sigma, 14533) dilute in ×1 PBS for 30 min at room temperature. For LipidTOX data in Figure 4e,f, measurements were pooled across three independent experimental replicates from at least four printed‐niche replicates in each experiment for a total of at least 24 fields‐of‐view per niche condition. In Figure 4k, LipidTOX measurements were pooled across three independent experimental replicates from at least four printed‐niche replicates in each experiment for a total of 12 bone‐fat niches. Following incubation, samples were washed an additional ×3 with ×1 PBS containing 0.05 w/v % sodium azide. Fluorescent microscopy of large fields of view (arrays of RGD and Young's modulus) was tiled using a Nikon Ni‐E microscope with a motorized stage, monochrome DS‐Qi2 camera, and Plan Apo Lambda 10×/0.45NA dry objective. Confocal microscopy was completed on a Zeiss LSM 800 Confocal microscope using ×63 Plan‐Apo 63×/1.40NA Oil objective (with an in‐plane lateral resolution of 0.413 µm per pixel), pinhole diameter of 1.0 AU (50.34 µm), and azimuthal resolution of 0.4 µm. Segmentation was performed using custom MATLAB scripting that makes use of the open microscopy Bio‐Formats tool. Masks were created to define cytoplasmic and fat volumes using phalloidin and LipidTOX stains. For production and purification of the fluorescent collagen 1A probe, the pET28a‐EGFP‐CNA35 plasmid was received as a gift from Maarten Merkx (Addgene plasmid # 61603; http://n2t.net/addgene:61603; RRID: Addgene_61603) and synthesized as reported previously.^[^ ^60^ ^]^ In brief, protein yields of the CNA35 probe were synthesized using E.Coli bacteria before purification using ÄKTApurifier (Cytiva) and a 5 ml Ni‐NTA Superflow Cartridge (Qiagen), dialysis with SnakeSkinTM Dialysis tubing with 10 kDa MWCO, and concentration with an Amicon 10 kDa MWCO centrifugal filter unit. For imaging, 0.5 µM of EGFP‐CNA35 solution was added to the sample and incubated on a plate rocker for 15 min, before washing twice with PBS. In Figure 4k, Col1A measurements were pooled across three independent experimental replicates from at least four printed‐niche replicates in each experiment for a total of 12 bone‐fat niches.

### Statistical Analysis

Wherever possible, a transparent interpretation of the data had been provided, showing each measurement as a “scatter‐dot” alongside its corresponding distribution in the form of a violin plot. The *p*‐values for all reported biological data are reported in Table S3, Supporting Information. By using these methods, the extent to which the dependent variables of a material property affect the corresponding independent variable of cell function could be ascertained, allowing the interpretation of subtle differences in the magnitude of a given effect. Where available, the exact sample size (*n*) for each experimental group had been provided either in the figure legend or directly in the figure. For all cell culture experiments, a minimum of three independent experimental replicates were completed for each printed fabrication condition and relevant stain unless otherwise stated. Notably, Figure 1h,i shows experiments completed just once. Experiments in Figure S5, Supporting Information were conducted in duplicate only. For the sample shown in Figure 1d–g, only two independent experimental replicates were fabricated, as this sample only served to illustrate the CMFL methodology – one was mounted for confocal microscopy (Figure 1d,e), and the other was analyzed with force spectroscopy (Figure 1e,f). All experiments containing expression‐mapped markers were completed from at least three independent experimental replicates, except for experiments analyzing the effects of different concentrations of BMP4 on pSMAD1 N:C (Figure 6e–g and Figure S24, Supporting Information) completed in two independent replicates. Further information detailing the number of replicates and their measurement is included in the relevant Experimental Section. To segment and calculate the nuclear to cytoplasmic intensity of fluorescent markers in single cells for YAP and RUNX2 and to embryonic marker intensity and location, Python and MATLAB scripting was used with an adaption of the 3D StarDist algorithm as indicated in methods and Supporting Information. For null hypothesis testing, one‐way ANOVA with Tukey posthoc tests was used and listed throughout with *p*‐values noted, for not significant “n.s.” – *p* > 0.05, or significant *p* < 0.05. All violin plots were shown with a large central dot indicating the mean, with 1st/3rd quartile lines extending from the mean dot. Analysis was completed using MATLAB (2020a) to fit force‐displacement curves of force spectroscopic data. For force spectroscopy, three independently fabricated experimental replicates of each niche condition were fabricated with at least four replicates of each material condition. No data exclusions were made for biological data and quantitative analysis, except force spectroscopic curves where data was rejected if discontinuities were present, corresponding to samples slipping and thus an incomplete indentation of the AFM tip. No blinding was completed during experimentation or analysis. For cell culture experiments, comparative niche conditions (i.e., differing RGD, BMP2, or stiffness) and comparative controls (i.e., mechano‐structured vs uniform) were batched together and cultured/analyzed concurrently using non‐biased software approaches. All scripts used for data analysis are available upon request to the corresponding authors. All representative images were selected according to their proximity to the mean data across all replicates.

## Conflict of Interest

The authors declare no conflict of interest.

## Author Contributions

P.L.H.N. conceived the work, designed the modified MCFL 3D‐printer device and method, designed, and executed all experimentation, analyzed all data, and wrote the manuscript. Q.Y. and P.O. assisted with manuscript preparation, and together with J.S., helped the development of methods relating to the hiPSCs. T.A. assisted with printing structures for hADSCs. D.K. prepared and provided CNA fluorescent probes and methods relating to their use. M.B. provided scientific oversight and assisted with the experimental analysis and manuscript preparation. P.P.L.T. funded experiments on stem cells, assisted experimental design and methodology of cell culture, provided scientific oversight, and assisted with manuscript preparation. J.‐W.S. funded and provided scientific oversight in developing methods for specifying niche mechanics and related force spectroscopic measurement. H.Z. funded the project, provided scientific oversight, and assisted with manuscript preparation.

## Supporting information

Supporting Information

## Data Availability

The data that support the findings of this study are available from the corresponding author upon reasonable request.
